# A case of acute lithium poisoning and hypermagnesemia involving advanced colon cancer-induced colonic obstruction

**DOI:** 10.1093/omcr/omae107

**Published:** 2024-09-12

**Authors:** Hideo Takayama, Takuya Komura, Taro Kawane, Toshiki Matsuo, Makiko Kimura, Masashi Nishikawa, Kiyoki Kitagawa, Wataru Omi, Kenichi Sakajiri, Ichiro Onishi, Satoru Sakagami, Taro Yamashita, Takashi Kagaya

**Affiliations:** Division of Gastroenterology, NHO Kanazawa Medical Center, 1-1 Shimoishibikimachi, Kanazawa, Ishikawa 920-0939, Japan; Division of Gastroenterology, NHO Kanazawa Medical Center, 1-1 Shimoishibikimachi, Kanazawa, Ishikawa 920-0939, Japan; Division of Gastroenterology, NHO Kanazawa Medical Center, 1-1 Shimoishibikimachi, Kanazawa, Ishikawa 920-0939, Japan; Division of Gastroenterology, NHO Kanazawa Medical Center, 1-1 Shimoishibikimachi, Kanazawa, Ishikawa 920-0939, Japan; Division of Gastroenterology, NHO Kanazawa Medical Center, 1-1 Shimoishibikimachi, Kanazawa, Ishikawa 920-0939, Japan; Division of Gastroenterology, NHO Kanazawa Medical Center, 1-1 Shimoishibikimachi, Kanazawa, Ishikawa 920-0939, Japan; Division of Internal Medicine, NHO Kanazawa Medical Center, 1-1 Shimoishibikimachi, Kanazawa, Ishikawa 920-0939, Japan; Division of Internal Medicine, NHO Kanazawa Medical Center, 1-1 Shimoishibikimachi, Kanazawa, Ishikawa 920-0939, Japan; Division of Internal Medicine, NHO Kanazawa Medical Center, 1-1 Shimoishibikimachi, Kanazawa, Ishikawa 920-0939, Japan; Division of Surgery, NHO Kanazawa Medical Center, 1-1 Shimoishibikimachi, Kanazawa, Ishikawa 920-0939, Japan; Division of Internal Medicine, NHO Kanazawa Medical Center, 1-1 Shimoishibikimachi, Kanazawa, Ishikawa 920-0939, Japan; Division of Gastroenterology, Kanazawa University, 13-1 Takaramachi, Kanazawa, Ishikawa 920-8641, Japan; Division of Gastroenterology, NHO Kanazawa Medical Center, 1-1 Shimoishibikimachi, Kanazawa, Ishikawa 920-0939, Japan

**Keywords:** lithium poisoning, hypermagnesemia, advanced colon cancer, colonic obstruction, hemodialysis, arrythmia

## Abstract

An 83-year-old woman presented with disturbance of consciousness and hand tremor. She had taken lithium carbonate 300 mg/day for bipolar disorder and magnesium oxide 660 mg/day for constipation. Blood tests revealed lithium poisoning, hypermagnesemia and acute kidney injury. Computed tomography showed colonic obstruction caused by cancer of the descending colon. In the outpatient section, her blood pressure decreased to 89/54 mmHg, and her heart rate dropped to 40 bpm. We considered that the obstructive ileus induced intravascular dehydration, which led to toxic serum concentrations of lithium and magnesium, triggering the emergence of severe arrythmia induced by sinus dysfunction. The patient was treated with fluid resuscitation and hemodialysis, followed by endoscopic stent replacement for the descending colon cancer obstruction. These treatments improved her general condition and alleviated the lithium poisoning, hypermagnesemia and colonic obstruction. Such a case is considered extremely rare.

## Introduction

Lithium carbonate is an important psychiatric agent used to treat bipolar disorder. However, lithium overdose, impaired renal function, concomitant medications can cause serious poisoning symptoms [1, 2]. Lithium toxicity is an important consideration in clinical practice because its therapeutic range is quite narrow (0.6–1.3 mmol/l) [1]. Preparations containing magnesium are also widely used in clinical practice, especially for constipation. Although cases of paralytic ileus in chronic lithium intoxication or hypermagnesemia have been reported [3, 4], we found no report of obstructive ileus which led to severe lithium toxicity or hypermagnesemia, according to a PubMed search using the terms *lithium poisoning* and *ileus* or the terms *hypermagnesemia* and *ileus*. Here, we report a rare and interesting case of acute lithium poisoning and hypermagnesemia that was associated with colon cancer-induced colonic obstruction.

## Case report

An 83-year-old woman became aware of hand tremors and nausea 3 days before admission. Considering the gradual onset of altered consciousness and immobility, she came to our hospital. She had taken lithium carbonate 300 mg/day for bipolar disorder and magnesium oxide 660 mg/day for constipation.

When she arrived to our hospital, her blood pressure was 157/139 mmHg, heart rate was 78 beats/min, regular. Her body temperature was 36.7°C. Her level of consciousness was consistent with a score of E4V4M6 on the Glasgow Coma Scale. The abdomen was distended but did not exhibit tenderness or rebound pain. No leg edema was observed, and there was no obvious paralysis of the extremities.

**Figure 1:**
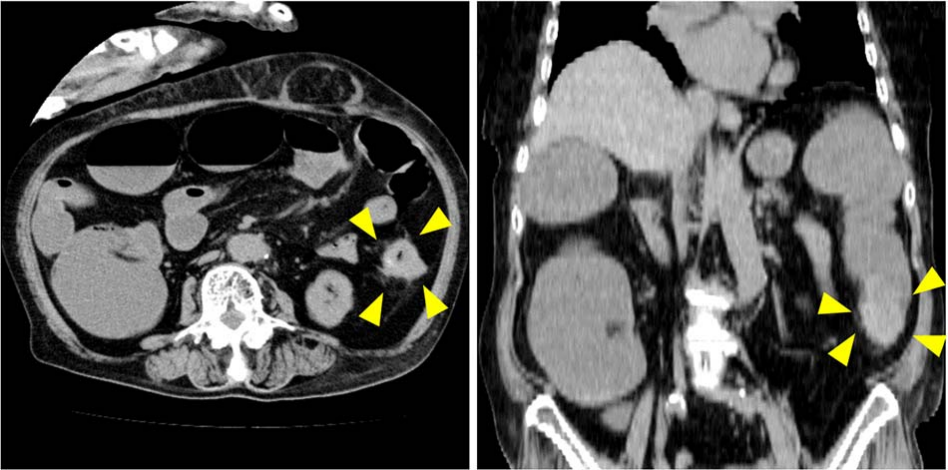
Computed tomography on arrival at our hospital revealed irregular wall thickening in the descending colon (arrow), accompanied by intestinal dilation proximal to the affected area. Obstructive ileus caused by cancer of the descending colon was suspected.

Table 1 shows the laboratory data on arrival. Blood chemistry tests revealed elevated concentrations of lithium (2.43 mmol/l), magnesium (6.1 mg/dl), and creatinine (2.26 mg/dl). The concentrations of the tumor markers CEA and CA19-9 were not substantially elevated. The result of the excretion of sodium (FENa) test was 0.1%, which indicated pre-renal dysfunction and suggested severe dehydration. Abdominal computed tomography (Fig. 1) reveals irregular wall thickening in the descending colon, with clinically significant dilation of the bowel proximal to this area and swelling of the regional lymph nodes. The patient was diagnosed with advanced cancer of the descending colon, which resulted in intestinal obstruction.

**Table 1:** Laboratory Data of Patients with lithium poisoning and hypermagnesemia on Arrival to Our Hospital

Hematology			Blood chemistry			Drug blood concentration		
WBC	20 300	/μl	T.Bil	0.6	mg/dl	Li	2.43	mmol/l
Neutro	85	%	AST	17	IU/l			
Lympho	7	%	ALT	8	IU/l	Blood tumor markers		
Mono	8	%	LDH	262	IU/l	CEA	3.4	ng/ml
RBC	412	×10^4/μl	ALP(IFCC)	172	IU/l	CA19-9	6.7	IU/ml
Hb	12.1	g/dl	Na	132	mEq/l			
Ht	38.2	%	K	5.0	mEq/l	Urine chemistry		
Plt	48.4	×10^4/μl	Cl	93	mEq/l	U-Na	9.0	mEq/l
			Ca	7.3	mg/dl	U-Cre	156	mg/dl
Blood coagulation tests			P	6.8	mg/dl			
PT	13	sec	Mg	6.1	mg/dl	The FENa test		
PT-INR	1.04		BUN	59.9	mg/dl	FENa	0.1	%
			Cr	2.26	mg/dl			
			TP	6.5	g/dl			
			Alb	3.4	g/dl			
			CRP	1.93	mg/dl			
			UA	16.1	mg/dl			
			HbA1c	5.2	%			

In the outpatient section, the patient’s blood pressure dropped to 89/54 mmHg, and her heart rate decreased to 40 beats/min. Based on the results of clinical examinations, we suspected hypovolemic shock due to intestinal obstruction and bradycardia due to lithium poisoning and hypermagnesemia. We promptly initiated fluid resuscitation and performed hemodialysis in the intensive care unit (ICU).

**Figure 2:**
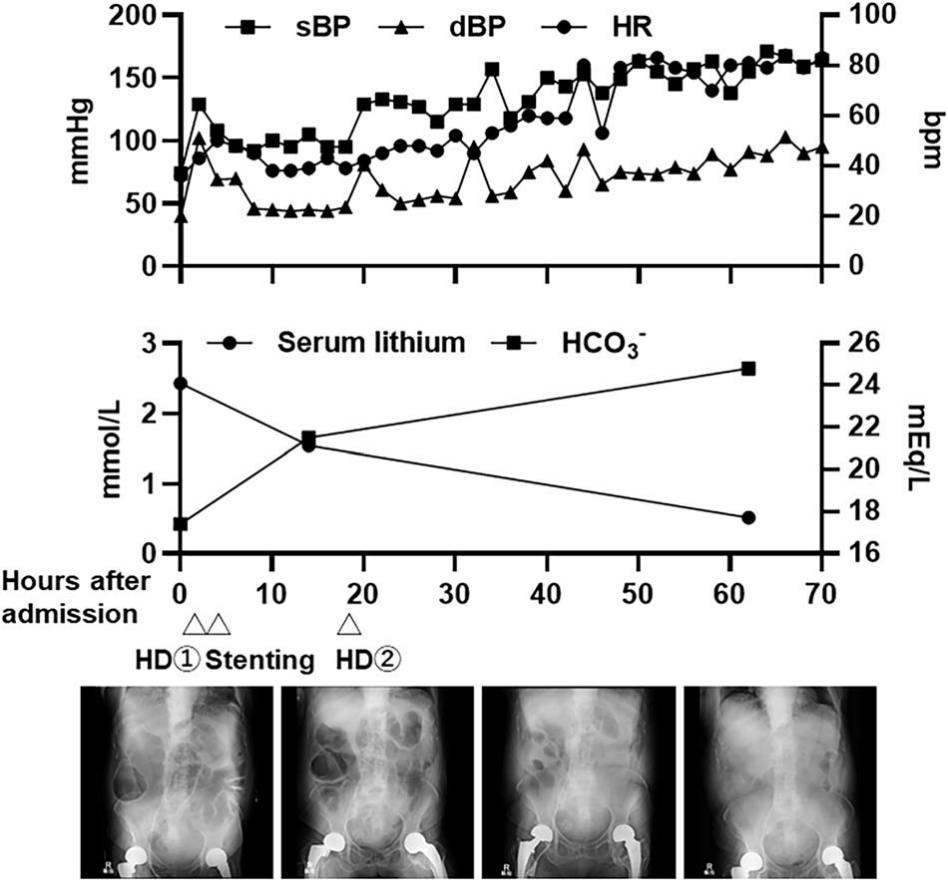
The patient’s clinical course in the ICU. Changes in vital signs, serum lithium concentration, HCO3- concentration, and X-ray findings. BP, blood pressure; HD, hemodialysis; HR, heart rate.

**Figure 3:**
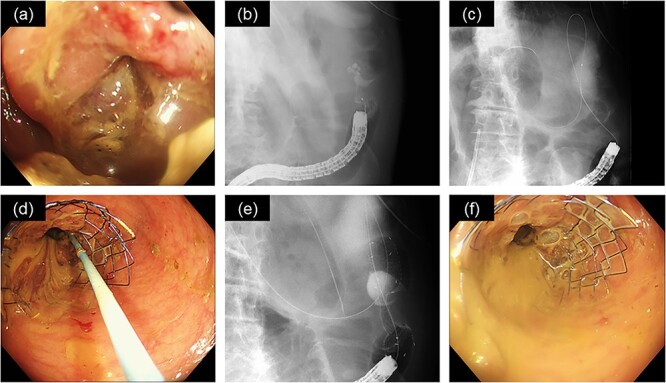
Endoscopic replacement of the colonic stent as treatment for cancer of the descending colon.

**Figure 4:**
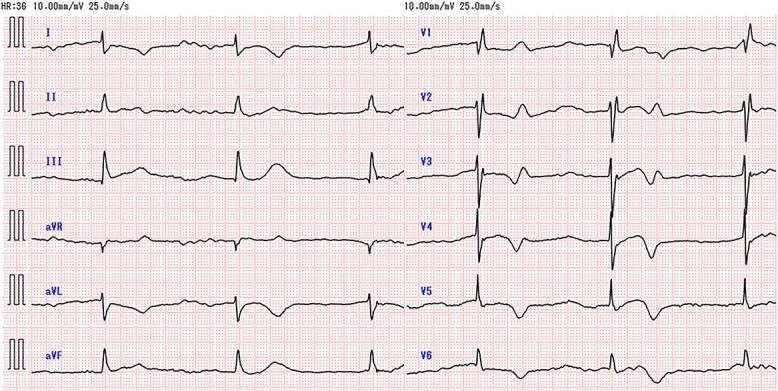
Electrocardiogram during bradycardia on admission day 2.

After admission to the ICU (Fig. 2), fluid resuscitation was continued, and emergency hemodialysis was performed to remove lithium and magnesium. The patient recovered from shock after these treatments. Emergency surgery to release the intestinal obstruction was hazardous because of the patient’s overall poor condition. Therefore, we performed endoscopic colonic stenting under X-ray guidance (Fig. 3). Endoscopic examination revealed a circumferential type 2 tumor in the descending colon, along with a stenosis that the endoscope could not pass (Fig. 3a). A smaller-diameter cannula and guidewire were passed through the stenosis (Fig. 3b and c); we also deployed an uncovered metallic stent (HANAROSTENT^®^ diameter 22 mm × length 9 cm; Boston Scientific, MA, USA) (Fig. 3d and e). Substantial flow of feculent material from the oral side of the intestine occurred after stent placement (Fig. 3f). After the procedure, we continued to manage her dehydration symptoms. On admission day 2, bradycardia occurred again, which was considered to be a result of lithium poisoning and hypermagnesemia-induced sinus dysfunction (Fig. 4). We considered temporary cardiac pacing but performed a second round of hemodialysis to alleviate lithium and magnesium toxicity. The patient’s vital signs gradually stabilized; her urine volume increased by admission day 4. The serum lithium concentration decreased to 0.51 mmol/l, and the serum magnesium concentration decreased to 2.6 mg/dl. Accordingly, no further hemodialysis was performed, and the patient was transferred to the general ward. Beginning on the day of transfer to the general ward, the patient resumed eating because her intestinal obstruction had been relieved. Rehabilitation was also conducted, and the patient was discharged to home on admission day 32.

The patient was later admitted to the surgical unit and underwent successful laparoscopic R0 resection of the descending colon. We diagnosed the patient with advanced cancer of the descending colon based on histopathology findings. The stage was determined as T4bN1aM0 (Stage IIIC) according to the TNM classification (Eighth Edition of the UICC).

## Discussion

Lithium carbonate serves as a key medication in the treatment of bipolar disorder and is used to augment antidepressants in patients with treatment-resistant depression [5]. However, lithium toxicity affects multiple organs, including the nervous, gastrointestinal, cardiovascular, and renal systems [1]. The severity of lithium toxicity ranges from mild to severe regardless of serum concentration. Although irreversible neurological symptoms have been reported in patients with severe lithium toxicity [2], the symptoms were reversible in our patient. Lithium-induced cardiotoxicity is rare, and reported effects include non-specific T-wave flattening, prolonged QT interval, sinus node dysfunction, ventricular tachycardia, cardiomyopathy, and myocardial infarction [6]. When diagnosing lithium toxicity, it is important to rapidly initiate intravenous fluid resuscitation to enhance renal elimination. Although the need for hemodialysis was unclear [1], the Extracorporeal Treatments in Poisoning (EXTRIP) Workgroup in 2015 recommended extracorporeal treatment in cases of severe lithium poisoning [7]. The recommendation includes when the patient exhibits decreased consciousness, or life-threatening arrythmias like this case.

Serum magnesium, along with lithium, is primarily excreted through the urine, and monitoring elevated blood levels is essential in kidney dysfunction patients. Generally, serum magnesium levels exceeding 7.2 mg/dl are associated with bradycardia and QT prolongation, while levels exceeding 12 mg/dl may cause atrioventricular block or cardiac arrest [8]. While severe bradycardia was observed in this case following admission, the serum magnesium concentration remained below 12 mg/dl. This indicates a potential risk of life-threatening arrhythmias at lower blood concentrations due to concurrent lithium toxicity.

In conclusion, colonic obstruction-induced acute lithium poisoning and hypermagnesemia is extremely rare; hemodialysis and endoscopic replacement of a colonic stent were effective management approaches for this case.
